# Supplementary material for: Multi‐dataset identification of innovative feature genes and molecular mechanisms in keratoconus
Source: J Cell Mol Med. 2024 Sep 19;28(18):e70079. doi: 10.1111/jcmm.70079 (PMC11412914; doi:10.1111/jcmm.70079)
Supplement: Supplementary file 2 — Material S2. [file JCMM-28-e70079-s002.zip › 7.GSEA analysis/2.ATOH7/KEGG_FC_EPSILON_RI_SIGNALING_PATHWAY.html]

Details for gene set KEGG\_FC\_EPSILON\_RI\_SIGNALING\_PATHWAY[GSEA]

|  || Dataset | ATOH7.group.cls#h\_versus\_l |
| Phenotype | group.cls#h\_versus\_l |
| Upregulated in class | h |
| GeneSet | KEGG\_FC\_EPSILON\_RI\_SIGNALING\_PATHWAY |
| Enrichment Score (ES) | 0.40238374 |
| Normalized Enrichment Score (NES) | 1.4224825 |
| Nominal p-value | 0.043396227 |
| FDR q-value | 0.29605156 |
| FWER p-Value | 0.885 |
Table: GSEA Results Summary

  

Fig 1: Enrichment plot: KEGG\_FC\_EPSILON\_RI\_SIGNALING\_PATHWAY      
 Profile of the Running ES Score & Positions of GeneSet Members on the Rank Ordered List

  

| SYMBOL | TITLE | RANK IN GENE LIST | RANK METRIC SCORE | RUNNING ES | CORE ENRICHMENT || 1 | AKT3 | na | 9 | 1.043 | 0.0892 | Yes |
| 2 | FYN | na | 470 | 0.560 | 0.1239 | Yes |
| 3 | PRKCE | na | 1002 | 0.447 | 0.1470 | Yes |
| 4 | FCER1A | na | 1210 | 0.419 | 0.1769 | Yes |
| 5 | CSF2 | na | 1491 | 0.387 | 0.2020 | Yes |
| 6 | PLA2G1B | na | 1671 | 0.371 | 0.2286 | Yes |
| 7 | IL3 | na | 1719 | 0.367 | 0.2588 | Yes |
| 8 | VAV1 | na | 1743 | 0.365 | 0.2894 | Yes |
| 9 | PLA2G2C | na | 2003 | 0.344 | 0.3114 | Yes |
| 10 | GAB2 | na | 2148 | 0.333 | 0.3358 | Yes |
| 11 | PRKCB | na | 2281 | 0.324 | 0.3598 | Yes |
| 12 | MS4A2 | na | 2640 | 0.301 | 0.3753 | Yes |
| 13 | PLA2G2A | na | 2876 | 0.287 | 0.3931 | Yes |
| 14 | MAP2K6 | na | 3338 | 0.263 | 0.4024 | Yes |
| 15 | MAP2K1 | na | 4240 | 0.228 | 0.3959 | No |
| 16 | PIK3R3 | na | 6166 | 0.166 | 0.3546 | No |
| 17 | LYN | na | 6447 | 0.158 | 0.3600 | No |
| 18 | MAPK12 | na | 6613 | 0.154 | 0.3685 | No |
| 19 | NRAS | na | 7693 | 0.126 | 0.3481 | No |
| 20 | PRKCA | na | 7938 | 0.120 | 0.3513 | No |
| 21 | FCER1G | na | 8619 | 0.106 | 0.3408 | No |
| 22 | BTK | na | 10420 | 0.075 | 0.2953 | No |
| 23 | GRB2 | na | 10441 | 0.075 | 0.3011 | No |
| 24 | LAT | na | 13347 | 0.037 | 0.2204 | No |
| 25 | RAC2 | na | 13916 | 0.031 | 0.2067 | No |
| 26 | TNF | na | 14399 | 0.027 | 0.1951 | No |
| 27 | MAPK1 | na | 14536 | 0.025 | 0.1933 | No |
| 28 | HRAS | na | 14559 | 0.025 | 0.1949 | No |
| 29 | RAF1 | na | 15219 | 0.019 | 0.1775 | No |
| 30 | PLA2G4A | na | 15227 | 0.019 | 0.1789 | No |
| 31 | MAP2K3 | na | 15568 | 0.016 | 0.1705 | No |
| 32 | AKT1 | na | 15787 | 0.014 | 0.1655 | No |
| 33 | PLA2G6 | na | 15813 | 0.014 | 0.1660 | No |
| 34 | PIK3CD | na | 16202 | 0.011 | 0.1557 | No |
| 35 | RAC1 | na | 16331 | 0.010 | 0.1529 | No |
| 36 | MAPK3 | na | 16821 | 0.006 | 0.1393 | No |
| 37 | MAPK8 | na | 17140 | 0.003 | 0.1304 | No |
| 38 | PRKCD | na | 17924 | -0.003 | 0.1081 | No |
| 39 | SYK | na | 18025 | -0.004 | 0.1056 | No |
| 40 | PLA2G12A | na | 18221 | -0.006 | 0.1005 | No |
| 41 | PLA2G2E | na | 18403 | -0.008 | 0.0959 | No |
| 42 | MAP2K7 | na | 18710 | -0.010 | 0.0880 | No |
| 43 | IL4 | na | 19594 | -0.018 | 0.0640 | No |
| 44 | MAP2K2 | na | 20653 | -0.028 | 0.0359 | No |
| 45 | PIK3CB | na | 20685 | -0.028 | 0.0374 | No |
| 46 | MAPK10 | na | 20989 | -0.032 | 0.0314 | No |
| 47 | VAV3 | na | 21180 | -0.034 | 0.0288 | No |
| 48 | MAP2K4 | na | 21265 | -0.035 | 0.0293 | No |
| 49 | PIK3CA | na | 21359 | -0.036 | 0.0297 | No |
| 50 | LCP2 | na | 21748 | -0.040 | 0.0220 | No |
| 51 | VAV2 | na | 22149 | -0.045 | 0.0142 | No |
| 52 | PIK3R2 | na | 22209 | -0.045 | 0.0164 | No |
| 53 | SOS2 | na | 22550 | -0.049 | 0.0108 | No |
| 54 | MAPK14 | na | 23324 | -0.059 | -0.0065 | No |
| 55 | JMJD7-PLA2G4B | na | 23573 | -0.063 | -0.0082 | No |
| 56 | MAPK13 | na | 23983 | -0.069 | -0.0140 | No |
| 57 | PLA2G3 | na | 24134 | -0.071 | -0.0122 | No |
| 58 | PDPK1 | na | 24308 | -0.074 | -0.0109 | No |
| 59 | PLCG2 | na | 24577 | -0.078 | -0.0119 | No |
| 60 | MAPK9 | na | 24826 | -0.082 | -0.0120 | No |
| 61 | KRAS | na | 25083 | -0.087 | -0.0119 | No |
| 62 | PLCG1 | na | 25238 | -0.090 | -0.0087 | No |
| 63 | MAPK11 | na | 25479 | -0.094 | -0.0076 | No |
| 64 | IL13 | na | 25720 | -0.099 | -0.0061 | No |
| 65 | PLA2G5 | na | 25954 | -0.103 | -0.0040 | No |
| 66 | PIK3CG | na | 26210 | -0.109 | -0.0020 | No |
| 67 | SOS1 | na | 26852 | -0.122 | -0.0100 | No |
| 68 | PLA2G10 | na | 27060 | -0.127 | -0.0051 | No |
| 69 | AKT2 | na | 27111 | -0.128 | 0.0044 | No |
| 70 | PLA2G4E | na | 27235 | -0.132 | 0.0122 | No |
| 71 | PLA2G2F | na | 27530 | -0.139 | 0.0156 | No |
| 72 | PIK3R1 | na | 27720 | -0.144 | 0.0225 | No |
| 73 | PLA2G2D | na | 28487 | -0.164 | 0.0144 | No |
| 74 | INPP5D | na | 28637 | -0.168 | 0.0245 | No |
| 75 | RAC3 | na | 32406 | -0.320 | -0.0568 | No |
| 76 | IL5 | na | 33446 | -0.410 | -0.0517 | No |
| 77 | PLA2G12B | na | 33562 | -0.422 | -0.0188 | No |
| 78 | PIK3R5 | na | 34383 | -0.611 | 0.0100 | No |
Table: GSEA details [plain text format]

  

Fig 2: KEGG\_FC\_EPSILON\_RI\_SIGNALING\_PATHWAY      
 Blue-Pink O' Gram in the Space of the Analyzed GeneSet

  

Fig 3: KEGG\_FC\_EPSILON\_RI\_SIGNALING\_PATHWAY: Random ES distribution      
 Gene set null distribution of ES for **KEGG\_FC\_EPSILON\_RI\_SIGNALING\_PATHWAY**
